# Fluid balance predicts weaning failure in chronic obstructive pulmonary disease patients

**DOI:** 10.1186/cc13489

**Published:** 2014-03-17

**Authors:** AC Pecanha Antonio, M Basso Gazzana, P Souza Castro, M Knorst

**Affiliations:** 1 Hospital Moinhos de Vento, Porto Alegre, Brazil; 2 UFRGS, Porto Alegre, Brazil

## Introduction

Fluid balance (FB) has been associated with weaning and extubation failure, particularly of cardiac origin. Also, the increased right ventricular afterload, a feature common in chronic obstructive pulmonary disease (COPD) patients especially during a weaning test, may hinder diastolic filling of the left ventricle through a biventricular interdependence mechanism. We aimed to investigate the relationship of the FB in the 48 hours prior to a spontaneous breathing trial (SBT) and weaning outcomes in a subgroup of COPD patients admitted to a medical-surgical ICU.

## Methods

We conducted a 2-year prospective, multicenter, observational study in two adult medical-surgical ICUs. All enrolled patients met eligibility criteria for ventilation liberation. Patients with tracheostomy were excluded. We collected demographic, physiologic, 48-hour FB (measured inputs minus outputs) and lung ultrasound findings immediately before a SBT in 29 COPD patients. Our main outcome of interest was weaning failure (WF), defined as the inability to tolerate a T-tube trial during 30 to 120 minutes, in which case patients were not extubated.

## Results

**Figure 1:**
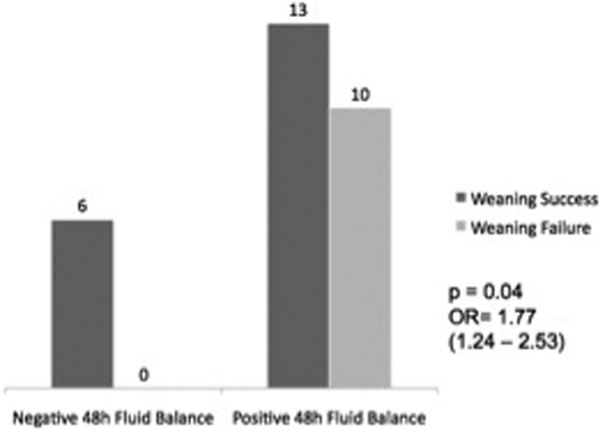
**Association between positive fluid balance WF in COPD patients**.

Weaning success (WS) (*n *= 19) and WF (*n *= 10) patients were similar in relation to age, sex, APACHE II score, reason for mechanical ventilation (MV) and comorbidities. Mean duration of MV was 11 days. FB in the 48 hours prior to the SBT did not differ between the Ws and WF groups (1,091.11 ± 2,195.89 ml and 2,398.80 ± 1,533.15 ml, respectively). Nevertheless, comparing individuals with 48-hour FB above and under the cutoff value of 0 ml according to weaning outcomes resulted in significant association between positive FB and WF in COPD patients (odds ratio = 1.77 (1.24 to 2.53)). That cutoff point was obtained on the ROC curve. See Figure 1.

## Conclusion

Positive FB in the 48 hours preceding the SBT predicted WF in COPD individuals. We recognized that no intervention was performed in order to accelerate the weaning process. Brain natriuretic peptide levels were not available.
